# Stress reactivity and pain‐mediated stress regulation in remitted patients with borderline personality disorder

**DOI:** 10.1002/brb3.909

**Published:** 2018-01-26

**Authors:** Franziska Willis, Sarah Kuniss, Nikolaus Kleindienst, Stefanie Lis, Janina Naoum, Martin Jungkunz, Corinne Neukel, Martin Bohus, Rolf‐Detlef Treede, Ulf Baumgärtner, Christian Schmahl

**Affiliations:** ^1^ Department of Psychosomatic Medicine and Psychotherapy Medical Faculty Mannheim Central Institute of Mental Health University of Heidelberg Mannheim Germany; ^2^ Department of General, Visceral and Transplantation Surgery University Hospital Heidelberg Heidelberg Germany; ^3^ Department of Psychosocial Medicine University of Heidelberg Heidelberg Germany; ^4^ Faculty of Health University of Antwerp Antwerp Belgium; ^5^ Department of Neurophysiology, Centre of Biomedicine and Medical Technology Mannheim Medical Faculty Mannheim Heidelberg University Mannheim Germany; ^6^ Department of Psychiatry, Schulich School of Medicine and Dentistry Western University London ON Canada

**Keywords:** borderline personality disorder, remission, self‐harm, stress regulation

## Abstract

**Objective:**

Patients with borderline personality disorder (BPD) use nonsuicidal self‐injury (NSSI) to cope with states of elevated inner tension. It is unclear to what extent remitted BPD patients experience these states and whether the experience of pain still regulates emotion. The purpose of this study was the investigation of baseline stress levels, stress reactivity, and pain‐mediated stress regulation in remitted BPD patients.

**Method:**

Subjective and objective stress parameters were assessed in 30 remitted BPD patients, 30 current BPD patients, and 30 healthy controls. After stress induction, a non‐nociceptive tactile stimulus, a tissue‐injuring, or a noninvasive pain stimulus was applied to the right volar forearm.

**Results:**

Baseline stress levels of remitted BPD patients lie in between the stress levels of current BPD patients and healthy controls. Urge for NSSI increased significantly more in current than remitted BPD patients. The experience of pain led to a greater decrease of arousal in current compared to remitted BPD patients and healthy controls.

**Conclusions:**

States of increased tension still seem to appear in remitted BPD patients. The role of pain‐mediated stress regulation appears to be reduced in remitted patients.

## SIGNIFICANT OUTCOMES

- Baseline stress levels of remitted BPD patients lie in between the stress levels of current BPD patients and healthy controls.
- Stress induction did not lead to a differential increase of arousal ratings or heart rate between current and remitted BPD patients and healthy controls; however, the increase of urge for NSSI was significantly larger in patients with current BPD.
- Immediately after the stimulus application, the experience of pain was associated with a larger decrease of arousal ratings in current than in remitted BPD patients.
- Higher pain experience was associated with lower arousal ratings in current BPD patients, whereas in remitted BPD patients and healthy controls, higher pain experience was associated with higher arousal ratings.

## Limitations

- Our study had a relatively small sample size and accordingly our results cannot be considered final and require replication.
- We compared the urge for NSSI between current and remitted BPD patients, even though inclusion criteria regarding the use of NSSI differed between the two groups. To create a more comparable situation between the two groups, these were matched according to urge for NSSI at baseline.
- To avoid a biased sample, we did not exclude patients with SSRI medication. SSRIs have emotion‐regulating effects and therefore may have influenced our results.

## 1 INTRODUCTION

In borderline personality disorder (BPD), emotion dysregulation is characterized by high baseline negative emotional intensity, high reactivity, and slow return to baseline (Linehan, 1993). Reflecting this dysregulated affect, patients with BPD experience states of high aversive inner tension (Stiglmayr, Shapiro, Stieglitz, Limberger, & Bohus, 2001). The termination of these states of arousal is the most prevalent motive for the use of nonsuicidal self‐injury (NSSI) and has been reported for 60%–90% of patients with BPD (Andover, 2014; Briere & Gil, 1998; Chapman, Gratz, & Brown, 2006; DiClemente, Ponton, & Hartley, 1991; Kleindienst et al., 2008; Klonsky, 2007; Paris, Brown, & Nowlis, 1987; Schoenleber, Berenbaum, & Motl, 2014; Zanarini et al., 2008). Most patients with BPD describe states of extreme aversive inner tension prior to acts of NSSI, and afterward feelings of relief and relaxation (Chapman et al., 2006; Kleindienst et al., 2008). Therefore, it has been suggested that NSSI reflects a dysfunctional attempt to cope with dysregulated affect (Niedtfeld & Schmahl, 2009; Reitz et al., 2015).

In patients with current BPD (BPD‐C), it was demonstrated that a tissue‐injuring pain stimulus (incision) leads to a reduction of stress indicated by both subjective (arousal ratings) and objective (heart rate) parameters (Reitz et al., 2012, 2015; Willis et al., 2016). Comparing current BPD patients with healthy controls (HC), there was a significantly greater decrease of stress after the incision stimulus in patients with BPD (Reitz et al., 2012, 2015). Further, a recent study revealed that stress reduction was achieved after both the application of an incision and a noninvasive pain stimulus suggesting that no tissue damage is necessary to reduce stress (Willis et al., 2016). On the neural level, the incision was followed by reduced amygdala activity and enhanced amygdala–prefrontal connectivity in BPD patients, suggesting that there is a link between pain perception and emotion regulation in BPD.

Remission and improvement of symptoms is a common phenomenon in BPD. (Gunderson et al., 2011; Zanarini, Frankenburg, Hennen, Reich, & Silk, 2006; Zanarini, Frankenburg, Hennen, & Silk, 2003; Zanarini, Frankenburg, Reich, & Fitzmaurice, 2016; Zanarini et al., 2007, 2012). A 16‐year follow‐up study reports that 99% of included BPD patients had 2‐year remissions and 78% had 8‐year remissions (Zanarini et al., 2012). The recurrence rates were 36% after a remission period of 2 years and 10% after a remission period of 8 years (Zanarini et al., 2012). Concerning NSSI, 97% of BPD patients had 2‐year remissions, and 91% of BPD patients had 4‐year remissions (Zanarini et al., 2016). Here, after 2‐year remissions of NSSI, the recurrence rate was 43%, and after 4‐year remissions, it was 33% (Zanarini et al., 2016). However, remitted BPD patients (BPD‐R) still show persistent impairment in social functioning (Gunderson et al., 2011). We do not know to what extent remitted BPD patients still experience states of elevated aversive inner tension. It is also unclear whether the association of pain perception with stress regulation still exists in remitted BPD patients.

We hypothesized that remitted BPD patients show lower stress levels than patients with current BPD, but still higher stress levels than healthy controls (I). In remitted BPD patients, we suspected a smaller increase of stress parameters compared to current BPD patients, but a smaller increase compared to healthy controls (II). Furthermore, we hypothesized that nociceptive stimuli will lead to a greater stress reduction in current BPD patients compared to remitted BPD patients and we tested if remitted BPD patients show a different response to nociceptive stimuli than healthy controls (III).

### 1.1 Aims of the study

To investigate whether states of high aversive inner tension still exist in patients with remitted BPD, whether stress reactivity differs between remitted and current BPD patients and healthy controls, and whether remitted patients are still able to regulate emotions with nociceptive experiences.

## 2 METHODS AND MATERIALS

### 2.1 Participants

From a larger, previously described sample (Willis et al., 2016), 30 female patients with current BPD and 30 female healthy controls (HC) were matched with a new group of 30 female remitted BPD patients according to age and educational background. Current and remitted BPD patients were additionally matched according to subjective ratings of urge for NSSI at the beginning of the experiment. Thus, participants did not significantly differ in age (BPD‐R: 28.97 [4.54], BPD‐C: 28.03 [6.07], HC: 28.73 [5.46] χ^2^ = 1.18 *df* = 2, *p *=* *.56), education (χ^2^ = 3.21 *df* = 2, *p *=* *.20), or urge for NSSI (BPD‐R: .12 [.41], BPD‐C: .23 [.39], *t*_(58)_ = 1.13, *p *=* *.26, *d *=* *.28).

For the group of current BPD patients, we only included patients who had shown NSSI with skin lesions at least once during the 6 months prior to study participation. Patients, who met the criteria for remission, were excluded if they had engaged in more than two acts of NSSI in the last 2 years; but all of them had used NSSI before. NSSI was assessed by a custom‐made questionnaire assessing the frequencies and forms of NSSI. The frequency and form of NSSI during the last month and the frequency of NSSI during the last year were evaluated (see Table 1).

**Table 1:** Sociodemographic data and pathology

	BPD remitted	BPD current	HC	*p*
Number	30	30	30	
Age (years)				
Mean (standard deviation)	29.0 (4.5)	28.0 (6.1)	28.73 (5.46)	.56^a^
Educational background				
University entrance diploma	22 (73%)	17 (57%)	23 (77%)	.20^b^
Secondary school certificate	8 (27%)	13 (43%)	7 (23%)	
Number of BPD criteria (current)				
Average number of criteria	.8 (1.1)	6.8 (1.2)	/	
0	16 (53%)	/	/	
1	6 (20%)	/	/	
2	5 (17%)	/	/	
3	3 (10%)	/	/	
4	/	/	/	
5	/	6 (20%)	/	
6	/	6 (20%)	/	
7	/	9 (30%)	/	
8	/	7 (23%)	/	
9	/	2 (7%)	/	
BPD criteria, current				
Frantic efforts to avoid abandonment	2 (7%)	15 (50%)	/	
Unstable, intense interpersonal relationships	2 (7%)	24 (80%)	/	
Identity disturbance	3 (10%)	20 (67%)	/	
Impulsivity in at least two potentially damaging areas	3 (10%)	16 (53%)	/	
Recurrent suicidal behavior, threats, gestures	1 (3%)	28 (93%)	/	
Affective instability	5 (17%)	29 (97%)	/	
Chronic feelings of emptiness	1 (3%)	26 (87%)	/	
Inappropriate, intense anger	3 (10%)	20 (67%)	/	
Paranoid ideation or dissociative symptoms	5 (17%)	25 (83%)	/	
Frequency of NSSI in the month before study participation				
Average frequency	.3 (.7)	16.1 (20.3)	/	
No NSSI in the month of study	28 (93%)	8 (27%)	/	
1–5 times	2 (7%)	6 (20%)	/	
6–10 times	/	2 (7%)	/	
11–20 times	/	1 (3%)	/	
21–30 times	/	6 (20%)	/	
More than 30 times	/	5 (17%)	/	
Unknown	/	2 (7%)		
Used methods of NSSI in the last year				
Cutting	1 (3%)	24 (80%)	/	
Scratching to the point of bleeding	/	19 (63%)	/	
Skin‐picking	1 (3%)	8 (27%)	/	
Self‐hitting	/	14 (47%)	/	
Burning/Scalding	/	10 (33%)	/	
Sticking needles or nails into skin	/	6 (20%)	/	
Hair tearing	/	8 (27%)	/	
Banging head against wall	/	9 (30%)	/	
Unknown	2 (7%)	/	/	

a Kruskal‐Wallis‐Test.

b Chi‐squared test.

Current BPD patients fulfilled at least five criteria for BPD diagnosis according to the Diagnostic and Statistical Manual of Mental Disorders (DSM‐5; APA, 2013). Remission was defined as no longer meeting a DSM‐5 diagnosis for BPD (Gunderson et al., 2011; Zanarini, Frankenburg, Reich, & Fitzmaurice, 2010; Zanarini et al., 2012, 2016). In our study, remitted BPD patients met no more than three criteria for BPD within the last 2 years, but had met the criteria for BPD at an earlier point in time (for details see Table 1). Borderline personality disorder (BPD) criteria were assessed via the International Personality Disorder Examination (IPDE) (Loranger, 1999).

Exclusion criteria for remitted and current BPD patients contained a lifetime diagnosis of bipolar I disorder or schizophrenia, mental retardation, a history of severe neurological dysfunction, the presence of severe psychopathology that required immediate treatment, and a current (past month) diagnosis of substance use disorder (including substance abuse and dependence). Patients with psychotropic medication were also excluded, except for those taking selective serotonin reuptake inhibitors (SSRIs) which were allowed (for current medication see Table S1). Co‐occurring psychiatric disorders were determined using the Structured Clinical Interview for Axis‐I Disorders (SCID‐I) (First, Spitzer, & Gibbon, 1995). Healthy controls were screened using the IPDE and SCID‐I as well. They were excluded if they met the diagnosis for any past or present psychiatric disorder or for substance abuse. All participants with a history of moderate‐to‐severe chronic pain, as well as participants with pain medication use in the 2 weeks prior to study participation, were excluded. For sociodemographic data and psychopathology, see Table S1.

Recruitment was performed by the central project of the KFO 256, a Clinical Research Unit funded by the German Research Foundation (DFG; KFO 256) dedicated to investigating mechanisms of disturbed emotion processing in BPD (Schmahl et al., 2014). Thus, all projects which originate from the KFO 256 include subjects from a joint database.

After having received a verbal and written explanation of the study procedure, all participants gave their written consent. The study was conducted according to the Declaration of Helsinki and approved by the ethics committee of the Medical Faculty Mannheim/University of Heidelberg (application no. 2008‐234N‐MA).

### 2.2 Experimental paradigm

#### 2.2.1 Stress induction

After a 3.5‐min baseline, stress was induced using a modified version of the Montreal Imaging Stress Task (MIST) (Dedovic et al., 2005), a generic stress task which induces stress in most subjects. Participants have to solve arithmetic tasks under time pressure. The program creates stress by manipulating both difficulty and time limit to simulate a poor performance. To add a social stress component, the participants' performance is displayed in relation to a fictitious average, and the investigator reminds the participants that the study depends on an above‐average performance.

#### 2.2.2 Nociceptive and tactile control stimuli

After the 30‐min stress induction, the participants were asked to put their right forearm behind a shield screen. After disinfection with alcohol (70%), balanced and randomized across groups either (1) a small incision was made (nociceptive with tissue injury), or (2) a blade stimulus not penetrating the skin (nociceptive without injury), or (3) a sham stimulus (non‐nociceptive; tactile) was applied. The incision stimulus was conducted according to the standardized incision protocol (Kawamata et al., 2002). With a sterile scalpel, a 4 mm long and 5–7 mm deep incision through skin, fascia, and muscle was performed. The small incision was well tolerated by all participants. The blade stimulator consisted of a blunt blade (tip dimensions 4.0 × 0.1 mm) attached to a plastic cylinder mounted with a weight that moves freely within a steel tube. With repeated application, exertion of the same force is ensured (4,096 mN; MRC Systems GmbH, Heidelberg, Germany). The blade stimulus was applied for 7‐s. For the sham stimulus, the forearm was touched with the scalpel grip, which evoked a slight sensation of touch. Until the stimulus was applied, participants were unaware of which stimulus to expect. The stimulus application was followed by a 31.5‐min relaxation phase.

#### 2.2.3 Dependent variables

As a subjective measure of stress, participants rated their current level of arousal on a visual analogue scale using Self‐Assessment Manikins (SAM) (Bradley & Lang, 1994). Participants rated arousal from 1 (relaxed) to 9 (under extreme tension) at 19 time points: before and after baseline (average score: mean‐baseline), seven times during stress induction, and ten times after stimulus application. Additionally, participants rated the urge for NSSI on a visual scale from 0 (none) to 10 (extreme) at the same time points. Directly after stimulus application, participants rated the pain intensity of the stimulus on a visual analogue scale from 0 (no pain) to 10 (worst imaginable pain).

**Figure 1:**
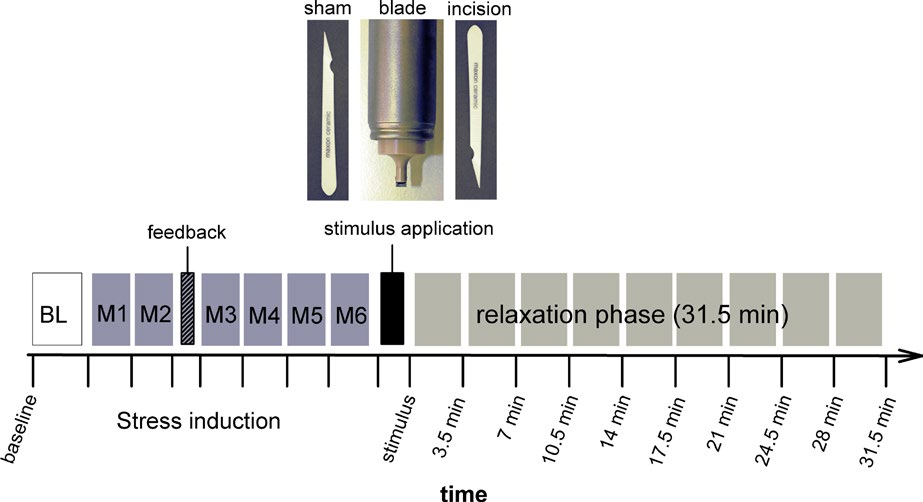
Study design: after a 3.5‐min baseline stress was induced with the MIST program (M1–M6). Then either the incision, blade, or sham stimulus was applied on the right volar forearm. The pain intensity of the stimulus was rated directly after the stimulus application. The stimulus application was followed by a relaxation phase. Current level of arousal, urge for NSSI, and heart rate was assessed throughout the experiment. This figure was modified from Willis et al. (2016)

In addition to subjective stress parameters, we continuously recorded heart rate as an objective measure of stress. We used ECG recording amplified with a BioSemi Active Two AD‐Box (Honsbeek, Kuiper & Van Rijn, Biosemi B.V., Amsterdam, the Netherlands) and reusable flat active Ag‐AgCl electrodes, digitized at 2 kHz. For the analysis, the experiment was split into 28 time points analogue to the subjective ratings: baseline (3.5 min), 18 time points during stress induction (1.2‐min intervals), and nine time points during the relaxation period (3.5‐min intervals). For study procedure, see Figure 1. See also (Willis et al., 2016) for a more detailed description of the study procedure.

At the beginning of the experiment, participants were informed that they would receive one of the three stimuli behind a shield screen so that they would not know which stimulus to expect until the application itself. Further, they were told that after the stimulus application, they will only have to rate their current arousal as well as their current urge for NSSI every 3.5 min for a total time of 30 min.

As reported in the above‐mentioned study (Willis et al., 2016), it appears likely that the stress‐reducing effect of the stimuli is caused by pain experience. Due to the smaller sample size and wider distribution of pain ratings for each stimulus in this sample (see Figure S1), in this study, not the stimulus type but the pain rating directly after stimulus application was treated as independent variable.

As dependent variables, we used (1) subjective levels of arousal (SAM ratings), (2) heart rate (as objective, neurophysiological measure of stress), and (3) urge for NSSI (ratings).

### 2.3 Data analysis

For the statistical analysis, SPSS (Version 22.0.0.0) was used. The level of statistical significance was set to *p *≤* *.05 (two‐tailed). For effect sizes, Cohen's *d* was reported for *t* test analyses, Cohen's *f* ^*2*^ for analyses of variance (ANOVAs), and *r* for hierarchical linear models (Rosenthal, 1994).

#### 2.3.1 Baseline stress levels and stress increase

To test whether the groups differ in baseline subjective arousal levels, a 3*2 repeated measure analysis of variance (rm‐ANOVA) with Group (BPD‐R vs. BPD‐C vs. HC) as between‐factor and Time (pre‐baseline vs. post‐baseline) was calculated. For baseline heart rate levels, an one‐way ANOVA was used.

To test stress reactivity (II), a 3*2 repeated measure analysis of variance (rm‐ANOVA) with Group (BPD‐R vs. BPD‐C vs. HC) as between‐factor and Time ([mean‐] baseline vs. poststress) as within‐factor was calculated for SAM ratings, urge for NSSI, and heart rate. As none of the HCs showed an urge for NSSI, the tests were only performed for BPD‐R and BPD‐C.

#### 2.3.2 Stress decreases and group comparisons

In line with our previous study comparing patients with current BPD and healthy controls (Willis et al., 2016), we used hierarchical linear models (HLM) to analyze the decrease of arousal, heart rate, and urge for NSSI directly (immediate effects) and 30 min (intermediate effects) after stimulus application.

For immediate effects, only the first time point directly after stimulus application and for intermediate effects, all time points after stress induction were analyzed.

To test to what extent pain experience leads to a reduction of stress parameters in patients with remitted BPD compared to current BPD patients and HC (III), both the effects of Pain intensity (pain rating) and Group (BPD‐R vs. BPD‐C vs. HC) were considered introducing both two‐way and three‐way interaction terms (Time*Group, Time*Pain Intensity, Group*Pain Intensity, Time*Group*Pain Intensity). As post hoc analyses HLMs with only two groups (BPD‐R vs. BPD‐C, BPD‐R vs. HC, and BPD‐C vs. HC) were performed. Again, analyses concerning urge for NSSI only included BPD‐C and BPD‐R. To prevent confounding by different levels of SAM, heart rate, and NSSI, baseline levels were used as an independent covariable.

The estimations in the linear hierarchical models were computed as maximum‐likelihood estimators using the MIXED procedure in SPSS.

## 3 RESULTS

### 3.1 Baseline levels and stress induction

**Figure 2:**
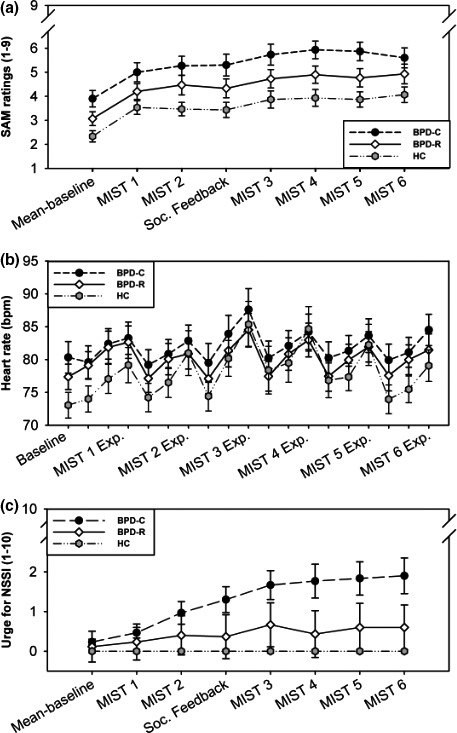
(a) Ratings of current level of arousal (SAM ratings) at baseline and during stress induction (MIST 1–MIST 6) among BPD‐C, BPD‐R, and HC. Arousal levels increased significantly in all groups. SAM ratings of BPD‐R lie in between the ratings of BPD‐C and HC. Error bars stand for the standard error of the mean (*SEM*). (b) Heart rate at baseline and during stress induction. The MIST software combines three different modes (rest, control, and experimental). During rest, no calculations have to be performed. During control and experimental modes, participants have to calculate during control without, and during experiment with a time limit. Heart rate levels of BPD‐R lie in between the heart rate levels of BPD‐C and HC. Heart rate increased significantly during stress induction in all groups. Error bars stand for the standard error of the mean (*SEM*). (c) Ratings of urge for NSSI at baseline and during stress induction (MIST 1–MIST 6). During stress induction urge for NSSI increased significantly more in BPD‐C compared to BPD‐R. Error bars stand for the standard error of the mean (*SEM*)

Concerning arousal, we found a significant difference in baseline levels between the three groups (*F*_2,87_ = 7.17, *p *=* *.001, *f* ^2^ = .16). The highest baseline arousal levels were found in BPD‐C, followed by BPD‐R, and HCs had the lowest baseline arousal levels (see Figure 2). In post hoc Bonferroni tests, there was a significant difference between BPD‐C and HC, but not between BPD‐R and BPD‐C, as well as between BPD‐R and HCs (BPD‐C vs. BPD‐R: *p *=* *.14; BPD‐R vs. HC: *p *=* *.24, BPD‐C vs. HC: *p *=* *.001). The same results were found for heart rate at baseline (*F*_2,87_ = 3.06, *p *=* *.05, Bonferroni: BPD‐C vs. BPD‐R: *p *=* *.94; BPD‐R vs. HC: *p *=* *.15, BPD‐C vs. HC: *p *=* *.02).

During stress induction, arousal levels significantly increased in all three groups, with BPD‐C showing the highest and HCs showing the lowest arousal levels (main effect *Time*:* F*_1,87_ = 91.14, *p* < .001, *f* ^2^ = 1.05; main effect *Group*:* F*_2,87_ = 6.58, *p *<* *.01, *f* ^2^ = .15; Bonferroni: BPD‐C vs. BPD‐R: *p *=* *.25; BPD‐R vs. HC: *p *=* *.10, BPD‐C vs. HC: *p *=* *.001). Heart rate increased in all three groups as well, but there was no significant difference between them (main effect *Time*:* F*_1,87_ = 28.68, p < .001, *f* ^2^ = .33; main effect *Group*:* F*_2,87_ = 2.38, *p *=* *.10, *f* ^2^ = .05).

We found no significant Time*Group interaction for arousal and heart rate during stress induction (all *p *>* *.05), indicating no difference in arousal and heart rate reactivity between the groups.

In contrast, regarding the urge for NSSI, there were differences between BPD‐R and BPD‐C. As the groups were matched for urge for NSSI at baseline, there was no significant difference in the beginning of the experiment (*F*_1,87_ = 1.29, *p* = .26, *f* ^2^ = .02). During stress induction, however, the urge for NSSI increased significantly stronger in BPD‐C (Time*Group:* F*_1,58_ = 5.80, *p* = .02, *f* ^2^ = .10). For baseline levels and stress increase of all three parameters see Figure 2.

### 3.2 Stress levels after pain stimulation

#### 3.2.1 SAM

**Figure 3:**
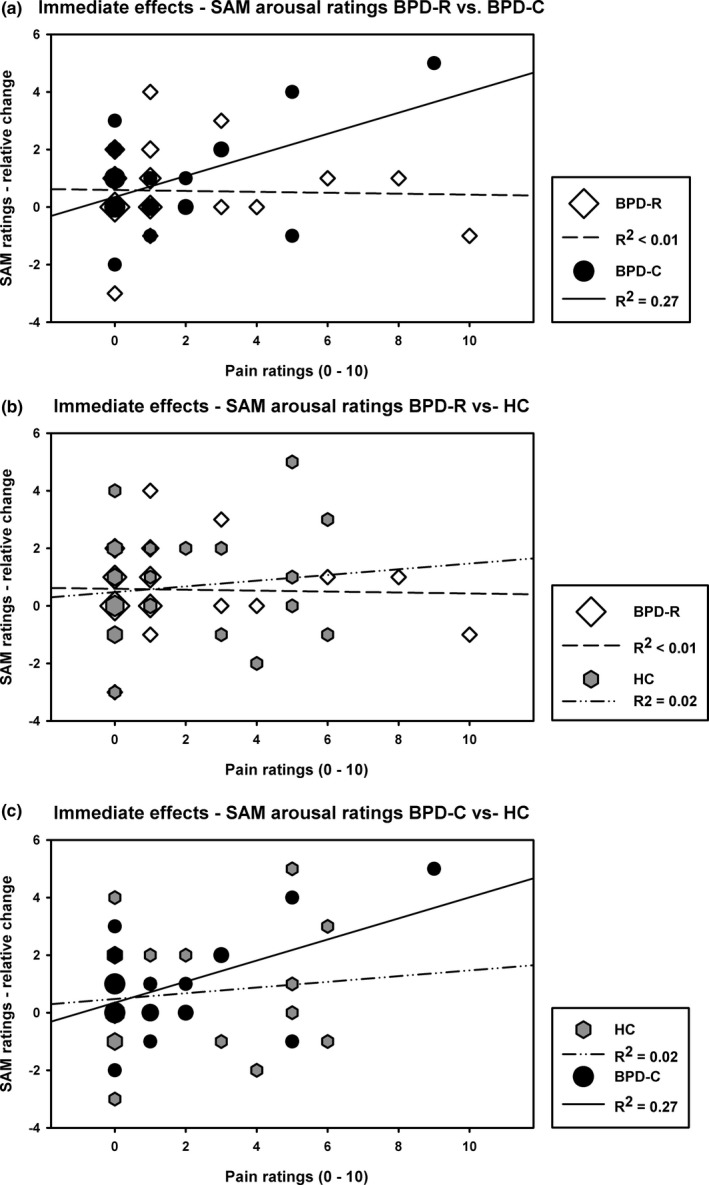
Immediate effects of stimulus application on arousal in BPD‐C, BPD‐R, and HC. Positive relative values for arousal change (arousal at stimulus application–MIST 6) reflect a decrease and negative values reflect an increase of arousal. Symbol size reflects the number of patients. (a) Arousal change in BPD‐R vs. BPD‐C directly after stimulus application with corresponding pain ratings reflecting the significant Time*Pain intensity*Group interaction (*p* = .01, *r* = .33). (b) BPD‐R and HC do not show a change in arousal depending on the pain intensity of the stimulus (c) Comparing BPD‐C to HC shows the same pattern as in (a) comparing BPD‐C to BPD‐R, but missed statistical significance

The HLM analyzing the behavior of SAM ratings dependent on the pain intensity of the stimulus directly after its application within all three groups showed a significant Time*Pain intensity interaction (β* *= −.42 [.21], *t *=* *5.01, *df *= 82, *p *=* *.05, *r *=* *.48), indicating that there is an association between pain experience and the course of arousal. A significant Time*Pain intensity interaction was also found comparing BPD‐R with BPD‐C (β = −.81 [.27], *t *=* *−3.06, *df *= 53, *p *=* *.003, *r *=* *.39), as well as BPD‐C with HC (β = −.55 [.22], *t *=* *−2.51, *df *= 56, *p *=* *.02, *r *=* *.32). Regarding all groups, there was no significant Time*Group*Pain intensity interaction (β = .13 [.10], *t *=* *1.38, *df *= 81, *p *=* *.17, *r *=* *.15). This effect was found, comparing BPD‐R to BPD‐C (Time*Pain Intensity*Group: β = .41 [.16], *t *=* *2.55, *df *= 53, *p *=* *.01, *r *=* *.33), indicating that only in BPD‐C a higher pain experience led to a greater decrease of arousal (see Figure 3a). The same pattern was found analyzing BPD‐C and HC (see Figure 3c), but missed statistical significance (β = 1.14 [.10], *t *=* *1.47, *df *= 56, *p *=* *.15, *r *=* *.19). Concerning BPD‐R and HC, no significant two‐ or three‐way interactions were found (all *p* > .05) (see Figure 3b).

**Figure 4:**
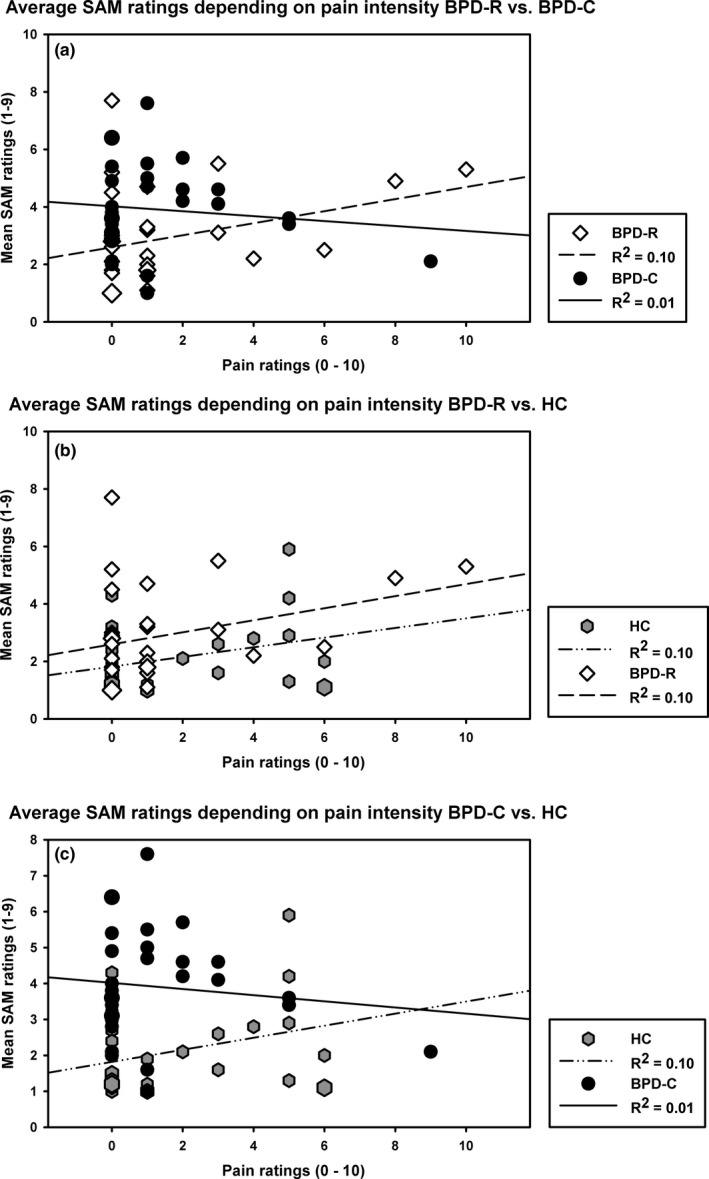
Mean levels of SAM ratings during the relaxation period in BPD‐C, BPD‐R, and HC (a) Mean of SAM ratings during the relaxation period depending on pain ratings in BPD‐C and BPD‐R. In BPD‐C, higher pain ratings are associated with lower SAM ratings reflecting the significant Pain intensity*Group interaction (*p* = .01, *r* = .28). (b) In both, BPD‐R and HC, higher pain ratings are associated with higher SAM ratings, and lower pain ratings are associated with lower SAM ratings. (c) Comparing BPD‐C to HC shows the same pattern as in (a) comparing BPD‐C to BPD‐R, but did not reach statistical significance

Considering the entire relaxation period, BPD‐C patients had significantly higher SAM ratings compared to BPD‐R patients (*Group*: β = −1.50 [.61], *t *=* *−2.47, *df *= 60, *p *=* *.01, *r *=* *.30) and compared to HC (β = −1.09 [.31], *t *=* *−3.51, *df *= 60, *p *=* *.001, *r *=* *.41). There was no main effect of *Group* comparing BPD‐R to HC (β = −.68 [.57], *t *=* *−1.21, *df *= 60, *p *=* *.23, *r *=* *.15). The HLM showed a significant Pain Intensity*Group interaction comparing BPD‐R and BPD‐C (β = .53 [.24], *t *=* *2.23, *df *= 60, *p *=* *.01, *r *=* *.28), indicating that greater pain experience was associated with higher SAM levels in the BPD‐R group, whereas in BPD‐C patients, greater pain experience was related to lower SAM ratings (see Figure 4a). This effect was also found regarding BPD‐C and HCs, but it did not reach statistical significance (β = .20 [.12], *t *=* *1.66, *df *= 60, *p *=* *.10, *r *=* *.21) (see Figure 4c). In both, BPD‐R and HC higher pain intensities were associated with higher arousal ratings (see Figure 4b).

#### 3.2.2 Heart rate

Directly after stimulus application, heart rate levels decreased in all three groups (*Time*: β = −43.83 [3.52], *t *=* *−12.46, *df *= 109, *p* < .001, *r *=* *.77). Heart rate decrease did not differ significantly between the groups and was not significantly related to pain perception (Time*Pain Intensity*Group: β = −.09 [.50], *t *=* *−.19, *df *= 118, *p *=* *.85, *r *=* *.02).The same results were found regarding the entire relaxation period (*Time*: β = −4.86 [.50], *t *=* *−9.75, *df *= 113, *p* < .001, *r* = .68; Time*Pain Intensity*Group: β = .09 [.06], *t *=* *1.49, *df *= 48, *p *=* *.14, *r *=* *.21).

#### 3.2.3 Urge for NSSI

Immediately after the stimulus application, there were no significant two‐ or three‐way interactions regarding the urge for NSSI. Ratings of urge for NSSI tended to be higher in the BPD‐C group, but this difference did not reach statistical significance (*Group*: β = −1.36 [.74], *t *=* *−1.85, *df *= 60, *p *=* *.07, *r *=* *.23). Regarding the entire relaxation period, BPD‐C patients showed significantly higher ratings of urge for NSSI than BPD‐R patients (*Group*: β = −1.33 [.55], *t *=* *−2.44, *df *= 60, *p *=* *.02, *r *=* *.30). The urge for NSSI significantly decreased in both groups, but the decrease was stronger in BPD‐C than BPD‐R (*Time*: β = −2.00 [.06], *t *=* *−3.06, *df *= 56, *p *=* *.003, *r *=* *.38; Time*Group: β = .08 [.04], *t *=* *2.07, *df *= 54, *p *=* *.04, *r *=* *.27). The pain intensity of the stimulus was largely unrelated to the decrease of urge for NSSI (Time*Pain Intensity: β = −.01 [.03], *t *=* *−.26, *df *= 54, *p *=* *.80, *r *=* *.04; Time*Pain Intensity*Group: β = .01 [.02], *t *=* *.42, *df *= 54, *p *=* *.68, *r *=* *.06).

For all two‐ and three‐way interactions see Tables [Link], [Link], [Link].

## 4 DISCUSSION

To our knowledge, this is the first study to investigate pain‐mediated stress regulation in remitted BPD patients. Our results suggest that tension levels of remitted BPD patients lie in between the levels of current BPD patients and healthy controls. The role of pain‐mediated stress regulation, however, appears to have evanesced in remitted BPD patients.

In our sample, remitted BPD patients seem to experience lower stress levels than current BPD patients, but still higher stress levels than healthy controls. There were no signs of increased stress reactivity between current and remitted BPD patients as well as healthy controls, as in response to the MIST stress paradigm, we found no difference in increase of stress parameters. However, remitted BPD patients still reacted with an increase of urge for NSSI during stress induction, even though acts of NSSI in this group were rare. Still, in current BPD patients, stress induction led to a significantly greater increase of urge for NSSI.

Before acts of NSSI, patients with BPD tend to experience high levels of aversive inner tension (Stiglmayr et al., 2005). Affect regulation is believed to be the strongest maintaining factor of NSSI and that the urge for NSSI is conditioned on aversive inner tension (Chapman et al., 2006; Klonsky, 2007). Our findings support these theories in current BPD patients, who develop an urge for NSSI during stress induction. However, the remitted BPD patients in our sample, who still seem to experience increased tension levels did not react with a similar increase of urge for NSSI. Our sample of current BPD patients regularly used NSSI, whereas the group of remitted patients barely did. We therefore propose that urge for NSSI is not only conditioned to the presence of aversive inner tension, but also that the regular use of NSSI reinforces itself and leads to an increased urge for NSSI during states of high aversive inner tension. Considering the concept of benign masochism (enjoying initially negative experiences after realization that the event is not threatening (Rozin, Guillot, Fincher, Rozin, & Tsukayama, 2013)), it could be possible that the more often NSSI is used the less threatening it is perceived. Whereas after a period of NSSI‐abstinence the threshold to use NSSI is higher. It appears likely that states with increased levels of aversive inner tension still exist in remitted BPD patients, but we do not know how they were able to cease the dysfunctional behavior of NSSI. It might be speculated that remitted BPD patients found other methods than NSSI to cope with elevated inner tension. These methods and the association of stress levels with completed treatments should be investigated in future studies.

Regarding self‐reported arousal, we could confirm our hypothesis that the experience of pain leads to a greater stress reduction in current compared to remitted BPD patients. As an immediate effect, the painfulness of the stimulus was correlated with arousal: The stronger the experience of pain, the more marked was the decrease of arousal ratings in current BPD patients. Comparing remitted BPD patients to healthy controls, in both groups, there were no signs of pain‐mediated stress regulation.

However, these results were not corroborated by the analysis of heart rate. Interestingly, several studies find discrepancies between subjective and objective measures of emotions in BPD (Krause‐Utz et al., 2013; Lampe et al., 2007; McCloskey et al., 2009; Willis et al., 2016). There might also be some more basic discrepancies between the assessment of emotions by questionnaires and by behavioral measures. BPD patients might tend to overrate emotional reactions or impulsivity on a psychometric level, which then cannot be completely verified in the laboratory. Also, the strong fluctuations of stress levels might lead to an overestimation of emotions or impulsivity. For future studies, here it might be helpful to additionally analyze heart rate variability and skin conductance to bridge this gap and add knowledge on the interaction of stress and emotions.

In line with our previous study (Willis et al., 2016), we did not find any longer lasting effects of the stimuli on stress decrease in any of the groups. Two recent studies suggest (Houben et al., 2017; Vansteelandt et al., 2017) that in BPD patients NSSI seems to help stabilizing negative affect rather than decreasing it. However, the above‐mentioned studies did not capture the immediate effects (seconds until minutes) directly following acts of NSSI. Therefore, it might be possible that NSSI has different short‐ and long‐term effects on stress regulation.

Still, there were differences concerning the reaction to pain experience concerning the 30‐min time interval succeeding the stimulus application. While among remitted BPD patients and healthy controls high pain intensity was associated with higher arousal ratings, the opposite pattern was observed for current BPD patients, where higher pain experience was associated with lower SAM ratings. This shows a difference in pain evaluation with remitted patients demonstrating a more normal correlation between pain experience and stress. These findings could, however, not be supported by the analysis of heart rate or urge for NSSI.

On a neurobiological level, incision is associated with reduced amygdala activity and improved amygdala–prefrontal connectivity in current BPD patients (Niedtfeld et al., 2012; Schmahl et al., 2006). In HCs, the opposite pattern was observed. This was interpreted as NSSI being a dysfunctional attempt to cope with dysregulated affect (Reitz et al., 2015). In the present study, we found that states of increased inner tension might still occur in remitted BPD patients, as they show stress levels between current BPD patients and healthy controls. However, the effects of nociceptive stimuli on stress regulation seem to have ceased, and the appraisal of pain appears to have normalized in remitted BPD patients. Whether on a neural level, the link between emotion regulation and pain perception is still present in remitted BPD patients or whether they show similar neural activation patterns to HCs should be investigated in future studies.

As a limitation, we would like to stress that our study had a relatively small sample size. For the main hypotheses, our study was adequately powered to detect medium to large effects (1‐β ≥.80; α = .05); however, due to the sample size, we may have missed smaller effects. Accordingly, our results cannot be final and conclusive and require further investigation. In this study, we compare the urge of NSSI between current and remitted BPD patients, even though inclusion criteria regarding the use of NSSI differed between the two groups. Frequent use of NSSI reflects the presence of severe dysfunctional behavior and is not consistent with our understanding of remission in BPD. However, we would like to stress that acts of NSSI and the urge for NSSI are not the same. To create a more comparable situation between the two groups, they were matched according to urge for NSSI at baseline. Furthermore, we did not exclude patients with SSRI medication. SSRIs have emotion‐regulating effects, which may have influenced the relation between dysregulated affect and pain perception. However, due to the high prevalence of psychotropic medication in BPD, the complete exclusion of psychotropic medication would have led to a biased sample. Furthermore, as already discussed elsewhere (Willis et al., 2016), after stress induction stress levels were only in a medium range, whereas before acts of NSSI BPD patients tend to experience higher tension levels (Stiglmayr et al., 2005).This might be related to the chosen stress induction, which limitedly considers components such as social rejection and the experience of shame, which are closely related to states of elevated inner tension in BPD patients (Chapman, Walters, & Dixon Gordon, 2014; Schoenleber et al., 2014). However, the strength of the MIST paradigm as generic stress induction is that it causes stress in most subjects.

Another difficulty discussing remission in BPD is the absence of a standard definition. Zanarini et al. (2003, 2008, 2012) define remission as no longer meeting five diagnostic criteria for BPD for 2 years, whereas for Gunderson et al. (2011), remission is defined as no longer meeting two or more BPD criteria for at least 12 months. As stated above, in our sample, remitted BPD patients did not meet more than three BPD criteria for at least 2 years. Further, fulfilling the remission criteria does not assess the functioning of the patients. Attaining good functioning is called recovery of BPD, which Zanarini et al. (2012) defined as a Global Assessment of Functioning (GAF) score higher than 60.

In our study, we only investigated two BPD symptoms, namely NSSI and tension/stress levels reflecting a dysregulated affect. But we did not assess the functioning of the patients on an everyday basis. Therefore, we cannot evaluate recovery of BPD in our sample of remitted BPD patients.

We found evidence for a fading association between nociception and tension relief, as well as for a reduced presence of urge for NSSI, and for a normalization of pain evaluation. For us, it is likely that these are important changes which might be necessary to recover from BPD.

In sum, we believe that our findings are an important step in the understanding of remitted BPD patients. But since our study was a pioneering study it awaits replication from an independent sample to confirm the present findings.

## CONFLICT OF INTEREST

F. W., S. K., N. K., S. L., J. N., M. J., C. N., M. B., R.‐D. T., U. B., and C. S. have no conflict of interest to declare.
