# Supplementary figures and images for: Stress reactivity and pain‐mediated stress regulation in remitted patients with borderline personality disorder
Source: Brain Behav. 2018 Jan 26;8(2):e00909. doi: 10.1002/brb3.909 (PMC5822574; doi:10.1002/brb3.909)

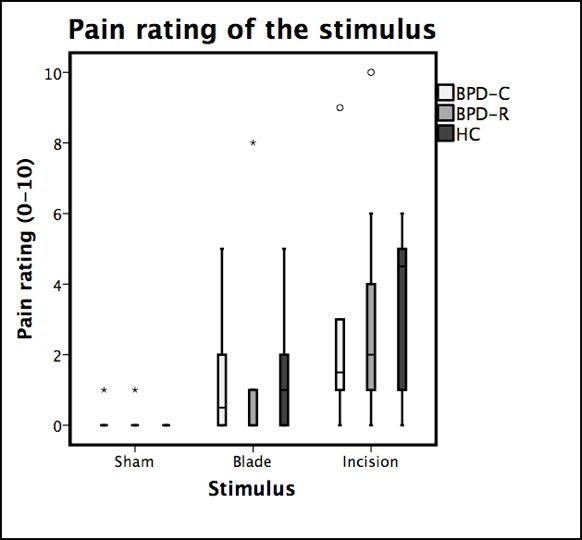

Supplement: Supplementary file 1 [file BRB3-8-e00909-s001.jpg]
