# Supplementary material for: Stress reactivity and pain‐mediated stress regulation in remitted patients with borderline personality disorder
Source: Brain Behav. 2018 Jan 26;8(2):e00909. doi: 10.1002/brb3.909 (PMC5822574; doi:10.1002/brb3.909)
Supplement: Supplementary file 2 [file BRB3-8-e00909-s002.docx]

**Table 1 (Supplement)**

|  | **BPD remitted** | **BPD current** | **HC** |
| --- | --- | --- | --- |
| **Psychiatric comorbidities** | | |  |
| Mood disorder, current  Mood disorder, lifetime | 2 ( 7%)  24 (80%) | 6 (20%)  25 (83%) | /  / |
| Substance abuse, lifetime | 11 (37%) | 12 (40%) | / |
| Substance dependence, lifetime | 7 (23%) | 9 (30%) | / |
| Anxiety disorder, current  Anxiety disorder, lifetime | 5 (17%)  14 (47%) | 12 (40%)  14 (47%) | /  / |
| Posttraumatic stress disorder, current  Posttraumatic stress disorder, lifetime | /  12 (40%) | 14 (47%)  16 (53%) | /  / |
| Obsessive-compulsive disorder, current  Obsessive-compulsive disorder, lifetime | 1 ( 3%)  2 ( 7%) | 3 (10%)  4 (13%) | /  / |
| Eating disorder, current  Eating disorder, lifetime | 2 ( 7%)  17 (57%) | 8 (27%)  14 (47%) | /  / |
| **Number of current diagnoses** | | |  |
| 0 | 19 (63%) | 4 (13%) | / |
| 1 | 8 (27%) | 12 (40%) | / |
| 2 | 1 ( 3%) | 9 (30%) | / |
| 3 | / | 2 ( 7%) | / |
| 4 | / | 3 (10%) | / |
| **Medication** | | |  |
| SSRI | 3 (10%) | 4 (13%) |  |
| Thyroid hormones | 4 (13%) | 4 (13%) | 4 (13%) |
| Oral contraceptives | 4 (13%) | 2 ( 7%) | 6 (20%) |
| Blood pressure lowering agents | / | 1 ( 3%) | / |
| Proton pump inhibitors | 1 ( 3%) | 1 ( 3%) | / |
| Antihistamine | / | 1 ( 3%) | / |
