# Supplementary material for: Stress reactivity and pain‐mediated stress regulation in remitted patients with borderline personality disorder
Source: Brain Behav. 2018 Jan 26;8(2):e00909. doi: 10.1002/brb3.909 (PMC5822574; doi:10.1002/brb3.909)
Supplement: Supplementary file 3 [file BRB3-8-e00909-s003.docx]

**Table 2: HLM for immediate effects**

| SAM ratings – all groups | | | | | |
| --- | --- | --- | --- | --- | --- |
|  | Parameter estimate  (mean ± standard error) | *df* | *t* | *p* | *r* |
| Time*Group | 0.13 (0.24) | 85 | 0.55 | p = 0.59 | 0.06 |
| Time*Pain Intensity | -0.42 (0.21) | 82 | -2.03 | **p = 0.05** | 0.22 |
| Group*Pain Intensity | -0.10 (0.18) | 90 | -0.54 | p = 0.59 | 0.06 |
| Time*Group*Pain Intensity | 0.13 (0.10) | 81 | 1.38 | p = 0.17 | 0.15 |
| Heart rate – all groups | | | | | |
|  | Parameter estimate  (mean ± standard error) | *df* | *t* | *p* | *r* |
| Time*Group | 0.87 (1.27) | 118 | 0.68 | p = 0.50 | 0.06 |
| Time*Pain Intensity | 0.37 (1.10) | 118 | 0.34 | p = 0.73 | 0.03 |
| Group*Pain Intensity | 0.02 (0.95) | 169 | 0.03 | p = 0.98 | <0.01 |
| Time*Group*Pain Intensity | -0.09 (0.50) | 118 | -0.19 | p = 0.85 | 0.02 |
| SAM ratings BPD-R vs BPD-C | | | | | |
|  | Parameter estimate  (mean ± standard error) | *df* | *t* | *p* | *r* |
| Time*Group | -0.06 (0.41) | 53 | -0.16 | p = 0.88 | 0.02 |
| Time*Pain Intensity | -0.81 (0.27) | 53 | -3.06 | **p < 0.01** | 0.39 |
| Group*Pain Intensity | -0.20 (0.35) | 60 | -0.58 | p = 0.56 | 0.16 |
| Time*Group*Pain Intensity | 0.41 (0.16) | 53 | 2.54 | **p = 0.01** | 0.33 |
| Heart rate BPD-R vs BPD-C | | | | | |
|  | Parameter estimate  (mean ± standard error) | *df* | *t* | *p* | *r* |
| Time*Group | 0.06 (2.34) | 76 | 0.25 | p = 0.80 | 0.03 |
| Time*Pain Intensity | -0.82 (1.52) | 76 | -0.54 | p = 0.60 | 0.06 |
| Group*Pain Intensity | -1.07 (1.73) | 114 | -0.62 | p = 0.54 | 0.06 |
| Time*Group*Pain Intensity | 0.73 (0.91) | 76 | 0.81 | p = 0.42 | 0.09 |
| Urge for NSSI BPD-R vs. BPD-C | | | | | |
|  | Parameter estimate  (mean ± standard error) | *df* | *t* | *p* | *r* |
| Time*Group | 0.24 (0.28) | 60 | 0.88 | p = 0.38 | 0.11 |
| Time*Pain Intensity | -0.03 (0.18) | 60 | -0.19 | p = 0.85 | 0.02 |
| Group*Pain Intensity | -0.05 (0.29) | 60 | -0.20 | p = 0.85 | 0.03 |
| Time*Group*Pain Intensity | 0.01 (0.11) | 60 | 0.10 | p = 0.92 | 0.01 |
| SAM ratings – BPD-R vs. HC | | | | | |
|  | Parameter estimate  (mean ± standard error) | *df* | *t* | *p* | *r* |
| Time*Group | 0.38 (0.52) | 53 | 0.73 | p = 0.47 | 0.10 |
| Time*Pain Intensity | -0.25 (0.47) | 52 | 0.53 | p = 0.60 | 0.07 |
| Group*Pain Intensity | 0.001 (0.32) | 60 | 0.003 | p = 1.00 | <0.01 |
| Time*Group*Pain Intensity | -0.13 (0.19) | 52 | -0.68 | p = 0.50 | 0.09 |
| Heart rate – BPD-R vs. HC | | | | | |
|  | Parameter estimate  (mean ± standard error) | *df* | *t* | *p* | *r* |
| Time*Group | 1.19 (2.44) | 66 | 0.49 | p = 0.63 | 0.06 |
| Time*Pain Intensity | 2.35 (2.19) | 66 | 1,07 | p = 0.29 | 0.13 |
| Group*Pain Intensity | 1.02 (1.70) | 111 | 0.60 | p = 0.55 | 0.07 |
| Time*Group*Pain Intensity | -0.85 (0.89) | 66 | -0.96 | p = 0.34 | 0.12 |
| Sam ratings – BPD-C vs. HC | | | | | |
|  | Parameter estimate  (mean ± standard error) | *df* | *t* | *p* | *r* |
| Time*Group | 0.15 (0.25) | 59 | 0.59 | p = 0.56 | 0.08 |
| Time*Pain Intensity | -0.55 (0.22) | 56 | -2.50 | **p = 0.02** | 0.32 |
| Group*Pain Intensity | -0.10 (0.19) | 60 | -0.55 | p = 0.58 | 0.07 |
| Time*Group*Pain Intensity | 0.14 (0.10) | 56 | 1.47 | p = 0.15 | 0.19 |
| Heart rate – BPD-C vs. HC | | | | | |
|  | Parameter estimate  (mean ± standard error) | *df* | *t* | *p* | *r* |
| Time*Group | 0.87 (1.35) | 85 | 0.65 | 0.53 | 0.07 |
| Time*Pain Intensity | 0.02 (1.22) | 85 | 0,02 | p = 0.99 | <0.01 |
| Group*Pain Intensity | -0.02 (0.98) | 114 | -0.02 | p = 0.98 | <0.01 |
| Time*Group*Pain Intensity | -0.09 (0.54) | 85 | -0.16 | p = 0.87 | 0.02 |
