# Supplementary material for: Stress reactivity and pain‐mediated stress regulation in remitted patients with borderline personality disorder
Source: Brain Behav. 2018 Jan 26;8(2):e00909. doi: 10.1002/brb3.909 (PMC5822574; doi:10.1002/brb3.909)
Supplement: Supplementary file 4 [file BRB3-8-e00909-s004.docx]

**Table 3: HLM for intermediate effects**

| SAM ratings – all groups | | | | | |
| --- | --- | --- | --- | --- | --- |
|  | Parameter estimate  (mean ± standard error) | *df* | *t* | *p* | *r* |
| Time*Group | 0.03 (0.03) | 73 | 0.83 | p = 0.41 | 0.10 |
| Time*Pain Intensity | 0.01 (0.03) | 70 | 0.47 | p = 0.64 | 0.06 |
| Group*Pain Intensity | 0.19 (0.12) | 90 | 1.58 | p = 0.12 | 0.16 |
| Time*Group*Pain Intensity | -0.01 (0.01) | 69 | -0.92 | p = 0.36 | 0.11 |
| Heart rate – all groups | | | | | |
|  | Parameter estimate  (mean ± standard error) | *df* | *t* | *p* | *r* |
| Time*Group | -0.10 (0.16) | 48 | -0.64 | p = 0.53 | 0.08 |
| Time*Pain Intensity | -0.19 (0.14) | 48 | -1.40 | p = 0.17 | 0.20 |
| Group*Pain Intensity | -0.91 (0.72) | 90 | -1.26 | p = 0.21 | 0.13 |
| Time*Group*Pain Intensity | 0.09 (0.06) | 48 | 1.49 | p = 0.14 | 0.21 |
| SAM ratings BPD-R vs BPD-C | | | | | |
|  | Parameter estimate  (mean ± standard error) | *df* | *t* | *p* | *r* |
| Time*Group | 0.05 (0.07) | 45 | 0.69 | p = 0.50 | 0.10 |
| Time*Pain Intensity | 0.05 (0.05) | 45 | 1.03 | p = 0.31 | 0.15 |
| Group*Pain Intensity | 0.53 (0.24) | 60 | 2.23 | **p = 0.03** | 0.28 |
| Time*Group*Pain Intensity | -0.04 (0.03) | 44 | -1.33 | p = 0.19 | 0.20 |
| Heart rate BPD-R vs BPD-C | | | | | |
|  | Parameter estimate  (mean ± standard error) | *df* | *t* | *p* | *r* |
| Time*Group | -0.08 (0.30) | 23 | -0.26 | p = 0.80 | 0.05 |
| Time*Pain Intensity | -0.28 (0.19) | 23 | -1.42 | p = 0.17 | 0.28 |
| Group*Pain Intensity | -0.37 (1.39) | 60 | -0.27 | p = 0.79 | 0.03 |
| Time*Group*Pain Intensity | 0.15 (0.12) | 23 | 1.31 | p = 0.20 | 0.26 |
| Urge for NSSI BPD-R vs. BPD-C | | | | | |
|  | Parameter estimate  (mean ± standard error) | *df* | *t* | *p* | *r* |
| Time*Group | 0.08 (0.04) | 54 | 2.07 | p = 0.04 | 0.27 |
| Time*Pain Intensity | -0.01 (0.03) | 54 | -0.26 | p = 0.80 | 0.04 |
| Group*Pain Intensity | 0.004 (0.21) | 60 | 0.18 | p = 0.97 | 0.02 |
| Time*Group*Pain Intensity | 0.01 (0.02) | 54 | 0.42 | p = 0.68 | 0.06 |
| SAM ratings – BPD-R vs. HC | | | | | |
|  | Parameter estimate  (mean ± standard error) | *df* | *t* | *p* | *r* |
| Time*Group | 0.01 (0.06) | 45 | 0.20 | p = 0.84 | 0.03 |
| Time*Pain Intensity | -0.05 (0.06) | 44 | -0.83 | p = 0.41 | 0.12 |
| Group*Pain Intensity | -0.12 (0.21) | 60 | -0.59 | p = 0.56 | 0.08 |
| Time*Group*Pain Intensity | 0.01 (0.02) | 44 | 0.48 | p = 0.63 | 0.07 |
| Heart rate – BPD-R vs. HC | | | | | |
|  | Parameter estimate  (mean ± standard error) | *df* | *t* | *p* | *r* |
| Time*Group | -0.09 (0.16) | 36 | -0.59 | p = 0.56 | 0.12 |
| Time*Pain Intensity | -0.22 (0.15) | 36 | -1.55 | p = 0.13 | 0.25 |
| Group*Pain Intensity | -0.90 (0.72) | 60 | -1.26 | p = 0.21 | 0.16 |
| Time*Group*Pain Intensity | 0.10 (0.06) | 36 | 1.55 | p = 0.13 | 0.25 |
| Sam ratings – BPD-C vs. HC | | | | | |
|  | Parameter estimate  (mean ± standard error) | *df* | *t* | *p* | *r* |
| Time*Group | 0.02 (0.03) | 51 | 0.69 | p = 0.49 | 0.10 |
| Time*Pain Intensity | 0.03 (0.03) | 48 | 0.95 | p = 0.35 | 0.14 |
| Group*Pain Intensity | 0.20 (0.12) | 60 | 1.66 | p = 0.10 | 0.21 |
| Time*Group*Pain Intensity | - 0.01 (0.01) | 47 | -1.08 | p = 0.28 | 0.16 |
| Heart rate – BPD-C vs. HC | | | | | |
|  | Parameter estimate  (mean ± standard error) | *df* | *t* | *p* | *r* |
| Time*Group | -0.08 (0.30) | 23 | -0.26 | p = 0.80 | 0.05 |
| Time*Pain Intensity | -0.28 (0.19) | 23 | -1.42 | p = 0.17 | 0.28 |
| Group*Pain Intensity | -0.37 (1.39) | 60 | -0.27 | p = 0.79 | 0.03 |
| Time*Group*Pain Intensity | 0.15 (0.12) | 23 | 1.31 | p = 0.20 | 0.26 |
