# Supplementary material for: Stress reactivity and pain‐mediated stress regulation in remitted patients with borderline personality disorder
Source: Brain Behav. 2018 Jan 26;8(2):e00909. doi: 10.1002/brb3.909 (PMC5822574; doi:10.1002/brb3.909)
Supplement: Supplementary file 5 [file BRB3-8-e00909-s005.docx]

**Table 4: HLM for immediate effects and immediate effects depending on the stimulus type**

| SAM ratings – immediate effects – all groups | | | | | |
| --- | --- | --- | --- | --- | --- |
|  | Parameter estimate  (mean ± standard error) | *df* | *t* | *p* | *r* |
| Time*Group | -0.12 (0.54) | 80 | -0.22 | p = 0.83 | 0.02 |
| Time*Stimulus | -0.62 (0.54) | 70 | -1.16 | p = 0.25 | 0.14 |
| Group*Stimulus | -0.22 (0.48) | 90 | -0.47 | p = 0.64 | 0.05 |
| Time*Group*Stimulus | 0.20 (0.25) | 79 | 0.82 | p = 0.42 | 0.09 |
| Heart rate – immediate effects – all groups | | | | | |
|  | Parameter estimate  (mean ± standard error) | *df* | *t* | *p* | *r* |
| Time*Group | 1.27 (2.77) | 120 | 0.46 | p = 0.65 | 0.04 |
| Time*Stimulus | 1.36 (2.75) | 120 | 0.49 | p = 0.62 | 0.04 |
| Group*Stimulus | -0.01 (2.39) | 171 | -0.002 | p = 1.00 | <0.01 |
| Time*Group*Stimulus | -0.25 (1.28) | 120 | -0.20 | p = 0.85 | 0.02 |
| Urge for NSSI ratings – immediate effects – BPD-C and BPD-R | | | | | |
|  | Parameter estimate  (mean ± standard error) | *df* | *t* | *p* | *r* |
| Time*Group | 0.85 (0.61) | 60 | 1.39 | p = 0.17 | 0.18 |
| Time*Stimulus | 0.32 (0.45) | 60 | 0.72 | p = 0.48 | 0.09 |
| Group*Stimulus | 0.90 (0.76) | 60 | 1.18 | p = 0.24 | 0.15 |
| Time*Group*Stimulus | -0.30 (0.28) | 60 | -1.06 | p = 0.29 | 0.14 |
| SAM ratings – immediate effects – all groups | | | | | |
|  | Parameter estimate  (mean ± standard error) | *df* | *t* | *p* | *r* |
| Time*Group | 0.004 (0.07) | 70 | 0.06 | p = 0.95 | 0.01 |
| Time*Stimulus | 0.004 (0.07) | 69 | 0.07 | p = 0.95 | 0.01 |
| Group*Stimulus | 0.22 (0.32) | 90 | 0.69 | p = 0.49 | 0.07 |
| Time*Group*Stimulus | 0.002 (0.03) | 69 | 0.07 | p = 0.94 | 0.01 |
| Heart rate – immediate effects – all groups | | | | | |
|  | Parameter estimate  (mean ± standard error) | *df* | *t* | *p* | *r* |
| Time*Group | 0.23 (0.34) | 49 | 0.67 | p = 0.51 | 0.10 |
| Time*Stimulus | 0.40 (0.34) | 48 | 1.17 | p = 0.25 | 0.17 |
| Group*Stimulus | -0.19 (1.80) | 90 | -0.11 | p = 0.92 | 0.01 |
| Time*Group*Stimulus | -0.10 (0.16) | 48 | -0.61 | p = 0.55 | 0.09 |
| Urge for NSSI ratings – immediate effects – BPD-C and BPD-R | | | | | |
|  | Parameter estimate  (mean ± standard error) | *df* | *t* | *p* | *r* |
| Time*Group | 0.13 (0.09) | 54 | 1.44 | p = 0.16 | 0.19 |
| Time*Stimulus | 0.06 (0.07) | 55 | 0.93 | p = 0.36 | 0.12 |
| Group*Stimulus | 0.47 (0.56) | 60 | 0.83 | p = 0.41 | 0.11 |
| Time*Group*Stimulus | -0.02 (0.04) | 54 | -0.43 | p = 0.67 | 0.06 |
